# Intratumoral T‐cell receptor repertoire is predictive of interim PET scan results in patients with diffuse large B‐cell lymphoma treated with rituximab/cyclophosphamide/doxorubicin/prednisolone/vincristine (R‐CHOP) chemoimmunotherapy

**DOI:** 10.1002/cti2.1351

**Published:** 2021-10-27

**Authors:** Mohamed Shanavas, Soi‐Cheng Law, Mark Hertzberg, Rodney J Hicks, John F Seymour, Zhixiu Li, Lilia Merida de Long, Karthik Nath, Muhammed B Sabdia, Jay Gunawardana, Maher K Gandhi, Colm Keane

**Affiliations:** ^1^ Mater Research University of Queensland Brisbane QLD Australia; ^2^ Department of Haematology Mater Hospital Brisbane QLD Australia; ^3^ Department of Haematology Prince of Wales Hospital and University of NSW Randwick NSW Australia; ^4^ Department of Cancer Imaging Peter MacCallum Cancer Centre East Melbourne Melbourne VIC Australia; ^5^ Department of Haematology Peter MacCallum Cancer Centre Royal Melbourne Hospital & University of Melbourne Parkville VIC Australia; ^6^ Centre for Genomics and Personalised Health School of Biomedical Sciences, Faculty of Health Translational Research Institute Queensland University of Technology (QUT) Woolloongabba QLD Australia; ^7^ Department of Haematology Princess Alexandra Hospital Brisbane QLD Australia

**Keywords:** immunotherapy, interim PET, lymphoma, TCR repertoire

## Abstract

**Objectives:**

A diverse intratumoral T‐cell receptor (TCR) repertoire is associated with improved survival in diffuse large B‐cell lymphoma (DLBCL) treated with rituximab/cyclophosphamide/doxorubicin/prednisolone/vincristine (R‐CHOP) chemoimmunotherapy. We explored the impact of intratumoral TCR repertoire on interim PET (iPET) done after four cycles of R‐CHOP, the relationships between intratumoral and circulating repertoire, and the phenotypes of expanded clonotypes.

**Methods:**

We sequenced the third complementarity‐determining region of TCRβ in tumor samples, blood at pre‐therapy and after four cycles of R‐CHOP in 35 patients enrolled in ALLGNHL21 trial in high‐risk DLBCL. We correlated the TCR diversity metrics with iPET status, gene expression profiles and HLA‐class I genotypes. We then sequenced the FACS‐sorted peripheral blood T cells in six patients, and pentamer‐sorted EBV‐specific CD8^+^ T cells in one patient from this cohort.

**Results:**

Compared with iPET^−^ patients, the intratumoral TCR repertoire in iPET^+^ patients was characterised by higher cumulative frequency of abundant clonotypes and higher productive clonality. There was a variable overlap between circulating and intratumoral repertoires, with the dominant intratumoral clonotypes more likely to be detected in the blood. The majority of shared clonotypes were CD8^+^ PD‐1^HI^ T cells, and CD8^+^ T cells had the largest clonal expansions in tumor and blood. In a patient with EBV^+^ DLBCL, EBV‐specific intratumoral clonotypes were trackable in the blood.

**Conclusion:**

This study demonstrates that clonally expanded intratumoral TCR repertoires are associated with iPET^+^ and that the blood can be used to track tumor‐associated antigen‐specific clonotypes. These findings assist the rationale design and therapeutic monitoring of immunotherapeutic strategies in DLBCL.

## Graphical abstract

In a cohort of high‐risk diffuse large B‐cell lymphoma patients enrolled in phase 2 clinical trial, clonally expanded intratumoral TCR repertoires were associated with a positive interim PET scans. Abundant intratumoral clonotypes were identified in peripheral blood at baseline and during chemoimmunotherapy.

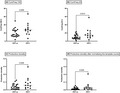

## Introduction

Diffuse large B‐cell lymphoma (DLCBL) is the commonest aggressive B‐cell lymphoma. It generally responds well to standard chemoimmunotherapy, with up to 65% of patients cured by their initial therapy.^1^ Unfortunately, one‐third of patients who are refractory to or relapse after the initial therapy have very poor outcomes.^2^, ^3^ Insights into the causes of these poor outcomes are vital to designing more effective and targeted treatment approaches.

Diffuse large B‐cell lymphoma employs a variety of immune evasion strategies associated with poor outcomes. These include the loss of antigen presentation molecules on the tumor cells and the facilitation of a tumor microenvironment (TME) that prevents effective tumor eradication.^4^, ^5^, ^6^ On account of these evasion strategies, T‐cell responses appear to play a critical role in determining the outcomes of the frontline treatment. It has previously been demonstrated that infiltration of CD4^+^ and CD8^+^ T cells within the TME is strongly predictive of improved outcomes to initial rituximab/cyclophosphamide/doxorubicin‐prednisolone/vincristine (R‐CHOP) chemoimmunotherapy.^7^, ^8^, ^9^, ^10^, ^11^, ^12^ However, the clonal structures and phenotypes of these intratumoral T cells in DLBCL are currently unknown.

The antigen specificity of T cells is determined by their TCR. Antigen experienced repertoires vary substantially in terms of TCR frequency and diversity. The complementarity‐determining region‐3 (CDR3) is the most variable region of the TCR and is critical for MHC‐peptide complex recognition. Because of this specificity, the TCRβ‐CDR3 sequence can be used as a ‘molecular tag’ to identify each T‐cell clone.^13^

In a previous work in DLBCL, we used high‐throughput sequencing of TCRβ‐CDR3 to establish that the less diverse TCR repertoires (measured as high TCR clonality) within the tumor biopsies characterised by high‐frequency clones were associated with inferior outcomes compared to repertoires with a broad range of T cells.^14^ This appeared to contrast with findings in solid organ tumors where a high TCR clonality appeared to predict improved outcomes. However, those observations were in a different context of immune checkpoint‐based therapy, whereas our findings were in DLBCL treated with chemoimmunotherapy.^15^

Although dynamic risk assessment using PET scan appears to predict outcomes in patients with DLBCL,^16^ there remain no data on the impact of the intratumoral TCR repertoire on iPET. There is also no current understanding whether intratumoral T‐cell responses are recapitulated in the systemic circulation in DLBCL, how these populations are influenced by chemoimmunotherapy, and whether peripheral blood can be used to track antigen‐specific intratumoral T‐cell populations. Our previous work was limited to tissue biopsies only and was unable to inform on the sharing of TCR repertoires between the tumor and the peripheral blood or on the phenotype of clonal T cells in the tumor microenvironment.

In the current study, we performed intratumoral TCR sequencing on diagnostic tissues donated by patients enrolled on a prospective phase II clinical trial, to examine the association of TCR clonality with results of interim PET scan after four cycles of R‐CHOP (iPET). In addition, we sought to discover whether TCR clonotypes in tumor biopsies at diagnosis could be identified in the peripheral circulation at diagnosis and whether they were altered under the influence of chemoimmunotherapy at the time of iPET restaging. We also sought to predict the phenotype of intratumoral clonotypes (ITC) indirectly from the phenotype of shared clonotypes identified in blood. Finally, as a proof of concept, employing EBV‐specific TCR clonotypes in a patient with EBV^+^ DLBCL, we sought to demonstrate that the antigen‐specific ITC can be tracked in peripheral blood.

## Results

### Patient characteristics

Among the 35 patients selected for the current analysis, 22 (63%) were iPET^−^ and 13 (37%) were iPET^+^. The median age was 56 years (range, 23–68), and 77% were males. IPI was low, intermediate and high in 15%, 64% and 21%, respectively, and 85% had advanced‐stage disease. The cell of origin (COO) was activated B cell (ABC) in 35%, germinal centre B cell (GCB) in 52% and unclassified (UNC) in 13%. These did not differ from the baseline characteristics of the complete clinical trial cohort from which these patients were selected: age (median 56 years vs 56 years, *P* = 0.7), sex (male 77% vs 66%, *P* = 0.2), IPI (group 0–1, 2–3, 4–5; 15%, 64% and 21% vs 17%, 62% and 21%, *P* = 0.9), stage (advanced stage, 85% vs 81%, *P* = 0.8) and iPET status (iPET^–^ 63% vs 74%, *P* = 0.3). Within the selected patients, the baseline characteristics were not significantly different between iPET^−^ and iPET^+^ patients (Table 1).

**Table 1:** Baseline characteristics of the 35 ALLGNHL21 patients who underwent TCR sequencing

	All (*n* = 35)	iPET^−^ (*n* = 22)	iPET^+^ (*n* = 13)	*P*
Age, median (range)	56 (23–68)	59 (23–68)	52 (36–66)	0.3
Sex, *n* (%)				
Male	27 (77%)	18 (82%)	9 (69%)	0.4
Female	8 (23%)	4 (18%)	4 (31%)	
IPI^a^, *n* (%)				
Low (0–1)	5 (15%)	2(10%)	3(23%)	0.2
Intermediate (2–3)	21 (64%)	12 (60%)	9 (69%)	
High (4–5)	7 (21%)	6 (30%)	1 (8%)	
Stage^a^, *n* (%)				
Early (1–2)	5 (15%)	2 (10%)	3 (23%)	0.4
Advanced (3–4)	28 (85%)	18(90%)	10(77%)	
COO^a^, *n* (%)				
ABC	8 (35%)	5 (36%)	3 (33%)	0.3
GCB	12(52%)	6 (43%)	6 (67%)	
UNC	3 (13%)	3 (21%)	0	

ABC, activated B cell; COO, cell of origin; GCB, germinal B‐cell; IPI, International Prognostic Index; UNC, unclassified.

^a^Missing or not available as follows: IPI (*n* = 2), stage (*n* = 2), COO (*n* = 12).

### Differential expansions of clonal intratumoral T cells between iPET^−^ and iPET^+^ patients

From the tumor biopsies, a median of 5400 (range, 603–48 501) total productive sequences (denotes the number of T cells sequenced) was obtained. Of these, 3172 (range, 447–19 934) were unique productive sequences (denotes distinct TCR clonotypes). The most abundant 100 clonotypes (CumFreq‐100) accounted for a median of 23.16% (range, 3.82–66.67) of total T cells sequenced. Similarly, the most abundant 25 clonotypes (CumFreq‐25) accounted for 11.10% (range, 1.49–57.69) of total T cells. The frequency of the most expanded clonotype (Max‐Freq) was a median of 1.55 (range, 0.12–10.26). The median productive clonality was 0.048 (range, 0.007–0.296; Table 2). As described in the Methods, a clonality score of 1 means only one clonotype was found in the examined repertoire, whereas a small score (e.g. 0.001) would indicate a significantly diverse TCR repertoire.

**Table 2:** TCR sequencing results in the tumor tissue, pre‐therapy and post‐cycle #4 blood samples

Sample	DNA input (ng)	Total productive sequences	Unique productive sequences	CumFreq‐100 (%)	CumFreq‐25 (%)	Max‐Freq (%)	Productive clonality
Tumor tissue	1611 (320–10 064)	5400 (603–48 501)	3172 (447–19 934)	23.16 (3.82–66.67)	11.10 (1.49–57.69)	1.55 (0.12–10.26)	0.048 (0.007–0.296)
PBMC Pre‐Rx	2495 (1240–2535)	46 512 (1 1189–218 764)	29 048 (7231–87 967)	19.31 (0.23–68.04)	15.09 (0.09–63.49)	3.02 (0.25–33.84)	0.09 (0.01–0.47)
PBMC post‐cycle #4	2493 (976–2520)	80 012 (1067–201 848)	27 799 (930–89 150)	18.30 (0.02–84.32)	12.36 (0.02–77.53)	2.56 (0.3–40.69)	0.10 (0.01–0.54)

Values are expressed as median (range).

CumFeq‐25, that of the most dominant 25 clonotypes; CumFreq‐100, the cumulative frequencies of the most dominant 100 clonotypes; Max‐Freq, the frequency of the most dominant clonotype, PBMC post‐cycle #4, peripheral blood mononuclear cells after four cycles of R‐CHOP; PBMC Pre‐Rx, peripheral blood mononuclear cells at diagnosis.

Next, the TCR repertoire was compared across tissue samples stratified by iPET status. Between iPET^+^ and iPET^−^ patients, there were no differences in gDNA input, median 1204 ng (range, 361–2948) vs 1935 ng (range, 320–10 064), (*P* = 0.2); total productive sequences, median 4768 (range, 874–48 501) vs 6307 (range, 603–34 428), (*P* = 1.0); or unique productive sequences, median 2867 (range, 756–18 571) vs 4046 (range, 447–19 934), (*P* = 0.5).

**Figure 1:**
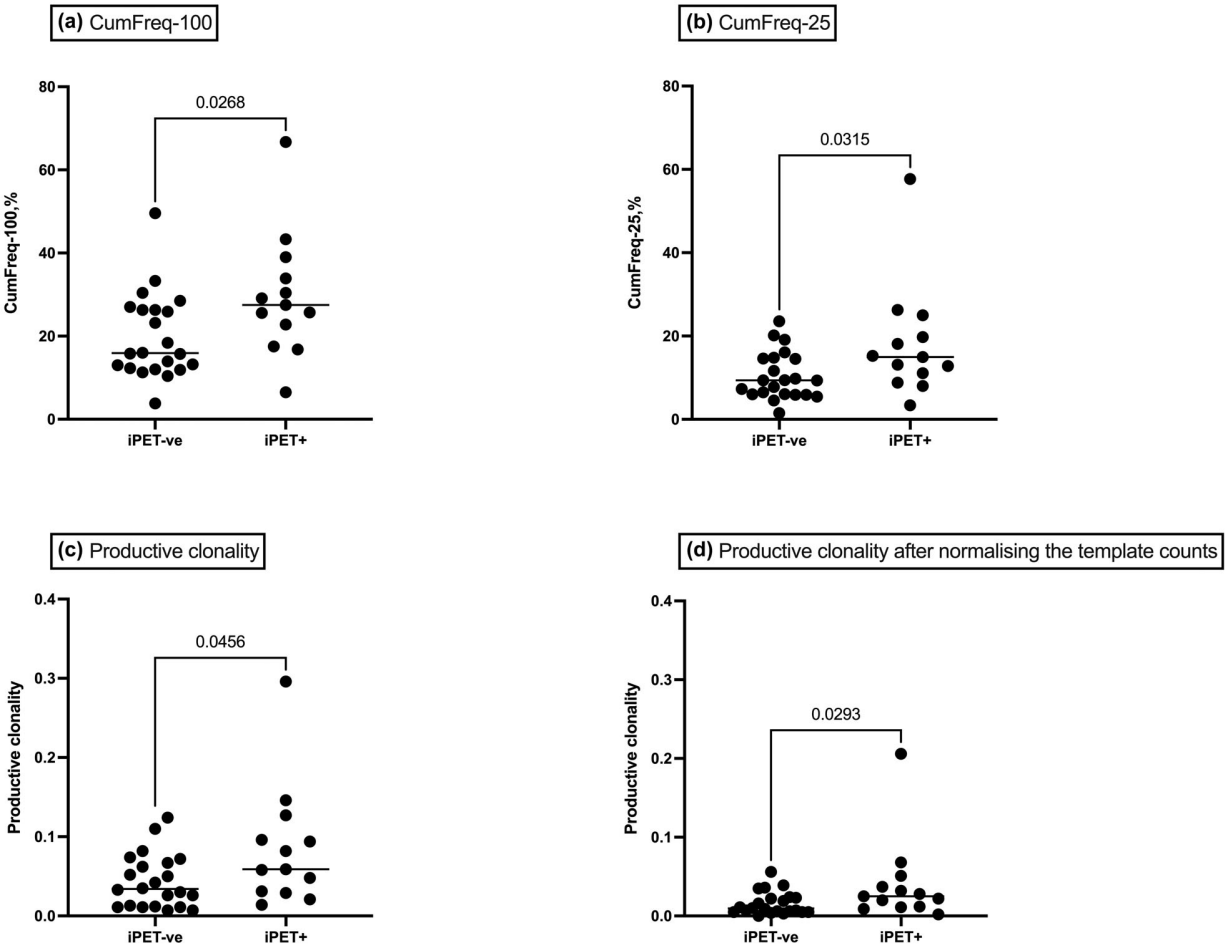
The TCR repertoires in DLBCL diagnostic biopsies stratified by iPET status. **(a)** Cumulative frequency of the most abundant 100 intratumoral TCR clonotypes (CumFreq‐100), **(b)** cumulative frequency of the most abundant 25 intratumoral TCR clonotypes (CumFreq‐25), **(c)** the productive clonality in the intratumoral TCR repertoire and **(d)** the productive clonality after downsizing by random drawing. In iPET^+^ patients, the CumFreq‐100 and CumFreq‐25 were higher suggesting a stronger level of oligoclonal expansion by high‐frequency clonotypes. The resulting high productive clonality was suggestive of lower clonotype diversity.

However, there were significant differences in CumFreq‐100 and CumFreq‐25 between iPET^+^ and iPET^−^ patients, suggestive of more high‐frequency clonotypes in iPET^+^ patients. The median CumFreq‐100 in iPET^+^ was 27.5% (range, 6.5–66.7%) vs 15.9% (range, 3.8–49.6%; *P* = 0.03); and median CumFreq‐25 was 14.95% (range, 3.36–57.69%) vs 9.35% (range, 1.49–23.55%; *P* = 0.03) respectively (Figure 1a and b). This also manifested as a higher productive clonality (a measure of lower TCR diversity) in iPET^+^ patients; median 0.059 (range, 0.014–0.296) vs 0.034 (range, 0.007–0.124; *P* = 0.046; Figure 1c). We cannot comment on the relative importance of iPET positivity in this cohort with regard to survival given treatment was subsequently stratified based on the iPET scan findings. There was no difference with regard to any of the TCR metrics and relative positivity of the PET scan (e.g. Deauville 4 vs Deauville 5) but large cohorts would likely be required to demonstrate such a finding. In addition, none of the clonality measurements correlated with measurements of tumor bulk as measured by stage of disease or LDH levels at diagnosis.

### Intratumoral T‐cell clones are detectable in the circulation at pre‐therapy and post‐cycle #4

The TCR repertoire was then assayed in the peripheral blood, so that the relationship between ITC and circulating clonotypes could be established. From pre‐therapy peripheral blood mononuclear cell (PBMC), a median of 46 512 (range, 11 189–218 764) of total productive sequences and 29 048 (range, 7231–87 967) of unique productive sequences was sequenced. The median CumFreq‐100, CumFreq‐25, Max‐Freq and the productive clonality were 19.31% (range, 0.23–68.04%), 15.09% (range, 0.09–63.49%), 3.02% (range, 0.25–33.84%) and 0.09 (range, 0.01–0.47), respectively (Table 2).

From post‐cycle #4 PBMC, a median of 80 012 (range, 1067–201 848) total productive sequences and 27 799 (range, 930–89 150) unique productive sequences were sequenced. The median CumFreq‐100, CumFreq‐25, Max‐Freq, and the productive clonality were 18.3% (range, 0.02–84.32%), 12.36% (range, 0.02–77.53%), 2.56% (range, 0.3–40.69%) and 0.10 (range, 0.01–0.54), respectively (Table 2). Clonality in the peripheral blood at diagnosis or after 4 cycles R‐CHOP was not associated with iPET results.

**Figure 2:**
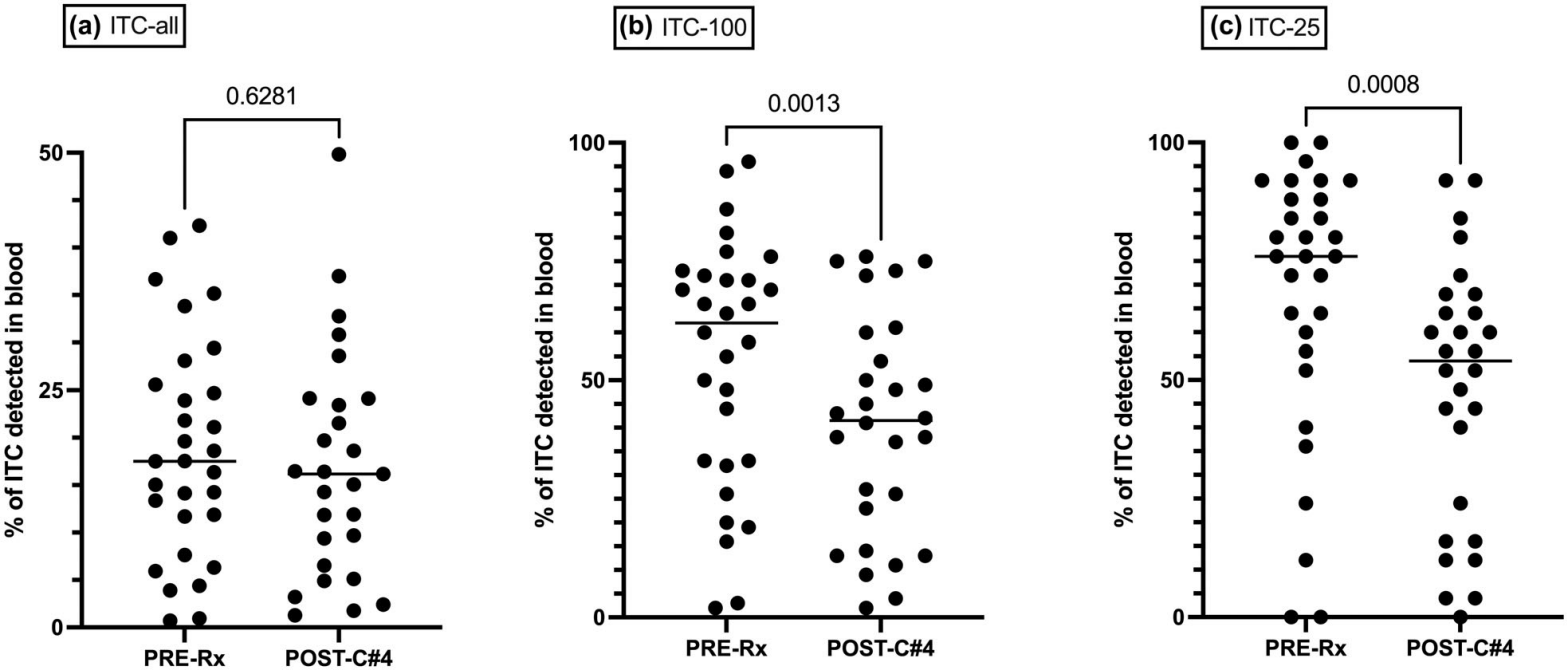
The dynamic changes in the intratumoral clonotypes (ITCs) detected in the peripheral blood during R‐CHOP, stratified by their abundance in the intratumoral repertoire. The proportion of ITCs detected in the peripheral blood at baseline (Pre‐Rx) and after four cycles of R‐CHOP (post‐c#4) are shown for **(a)** all ITCs (ITC‐all), **(b)** the most abundant 100 ITCs (ITC‐100) and **(c)** the most abundant 25 ITCs (ITC‐25). Although a greater proportion of ITC‐100 and ITC‐25 were detected in peripheral blood, there was significant reduction during treatment.

The proportion of ITC detected in PBMC was calculated separately for ITC in general (ITC‐all), as well as for the most abundant 100 ITC (ITC‐100) and most abundant 25 ITC (ITC‐25), and we analysed whether there was any difference between pre‐therapy and post‐cycle #4. For ITC‐all, a median 17.52% (range, 0.72–42.33%) was detected in pre‐therapy PBMC vs 16.17% (range, 1.27–49.83%) in post‐cycle #4 PBMC (*P* = 0.6). However, for more abundant ITC, a greater proportion was detected in PBMC, and at the same time, a more significant reduction was observed at post‐cycle #4. A median 62.0% (range, 2.0–96.0%) of ITC‐100 was detected at pre‐therapy but only 41.5% (range, 2.0–76.0%) at post‐cycle #4 (*P* = 0.001). The proportion detected in PBMC rose further when the ITC‐25 was interrogated, a median 76.0% (range, 0–100%) at pre‐therapy but reducing to 54.0% (range, 0–92%) at post‐cycle #4 (*P* < 0.001). Collectively, the data demonstrate that only those intratumoral T cells that have undergone large clonal expansions are detectable in the circulation. Furthermore, the proportion of dominant ITC detected in blood was highest at diagnosis and decreased after 4 cycles of R‐CHOP chemoimmunotherapy (Figure 2).

We then analysed whether the reduction in the expanded ITC during treatment was different between iPET^−^ and iPET^+^ patients. At baseline, the proportion of ITC‐100 detected in peripheral blood was median 54% (range, 2–96%) for iPET^−^ and 63% (range, 20–94%) for iPET^+^. After 4 cycles of R‐CHOP, this has decreased to 38% (range, 2–76%) in iPET^−^ and 49% (range, 4–79%) in iPET^+^. Wilcoxon matched pairs signed ranked test *P*‐values were 0.053 and 0.012, respectively, for iPET^−^ and iPET^+^. At either time points, the difference between iPET^−^ and iPET^+^ was not statistically significant (*P* = 0.4 and 0.2). In summary, the reduction of circulating tumor clonotypes during treatment was seen in both iPET^−^ (borderline *P*‐value) and iPET^+^ patients.

The ITC‐all contributed to a median of 20.15% (range, 0.1–70.3%) of all sequenced T cells in the blood at pre‐therapy and 10.9% (range, 0.6–70.7%) at post‐cycle #4 (*P* = 0.16). The ITC‐100 contributed to a median of 2.7% (range, 0–54.9%) of all sequenced T cells in the blood at pre‐therapy and decreased to 1.9% (range, 0–48.6%) at post‐cycle #4 (*P* = 0.02). Lastly, the ITC‐25 contributed to a median of 1.3% (range, 0–29.6%), of all sequenced T cells in the blood at pre‐therapy, and decreased to 0.6% (range, 0–11.7%) post‐cycle #4 (*P* = 0.02).

### High‐frequency intratumoral clonotypes are principally CD8^+^ PD‐1^HI^ T cells

Having demonstrated that there was sharing of T‐cell clonotypes between tumor and blood, we next used this to infer the phenotype of high‐frequency ITC in six patients who had sufficient pre‐therapy PBMC available for FACS sorting. The PBMC was sorted into seven populations and TCR sequencing was performed. The yield and TCR sequencing results of these phenotype populations are shown in Supplementary table 1.

**Figure 3:**
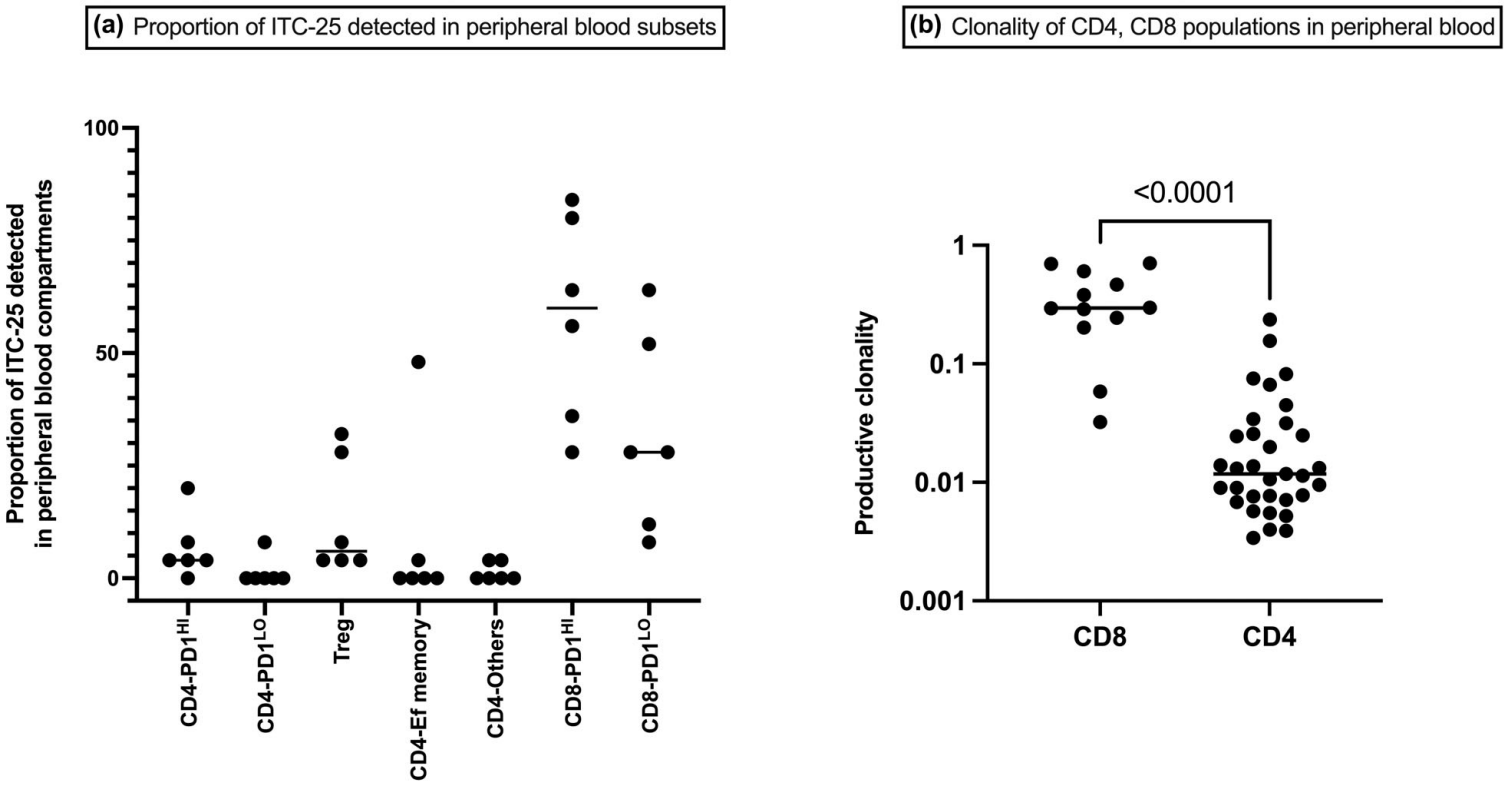
The proportion of ITC‐25 detected in peripheral blood T‐cell subsets, and the productive clonalities of CD4 and CD8 subsets in pre‐therapy blood. Pre‐therapy peripheral blood T cells were FACS sorted into seven subsets, and the clonotypes were matched with ITC from (unsorted) DLBCL diagnostic biopsies. **(a)** The proportion of the most abundant 25 intratumoral clonotypes (ITC‐25) detected in various peripheral blood T‐cell subsets are shown. The majority of ITC 25 were seen in CD8^+^ compartments in peripheral blood. **(b)** The productive clonalities of circulating CD4^+^ and CD8^+^ T cells. Here, the productive clonality of CD8^+^ T cells was significantly higher than that of CD4^+^ T cells.

The phenotypes of individual ITCs were then inferred from the phenotype of the shared clonotypes in the peripheral blood. With this method, the phenotype of median 17% (range, 10–25%) of ITC‐all, 50% (range, 27–73) of ITC‐100, and 74% (range, 44–92%) of ITC‐25 were predicted. Results showed that the vast majority of high‐frequency ITC were CD8^+^ T cells (Figure 3a). The majority of these were CD8^+^ PD‐1^HI^ and, to a lesser extent, were CD8^+^ PD‐1^LO^. Within ITC‐25, a median 60% (range, 28–84%) and 28% (range, 8–64%) were detected in the peripheral blood CD8^+^ PD‐1^HI^ and CD8^+^ PD‐1^LO^ T‐cell subsets, respectively. In comparison, only a small proportion of ITC‐25 were detected in CD4^+^ populations in the peripheral blood: in Treg, a median 9.8% (range, 5.6–34.8%); in CD4^+^ PD‐1^HI^, a median of 7.9% (range, 0–27.8%); in CD4^+^ PD‐1^LO^, a median of 0% (range, 0–11.1%); in CD4^+^ T effector memory cells, a median of 0% (range, 0–6.7%); and in the remaining CD4^+^ populations, a median of 0% (range, 0–5.6%).

In the pre‐therapy blood, CD8^+^ subpopulations were significantly more clonal than CD4^+^ subpopulations: median productive clonality 0.30 (range, 0.03–0.70) for combined CD8^+^ T‐cell subsets vs 0.01 (range, 0.003–0.23) for combined CD4^+^ subsets (*P* < 0.0001) (Figure 3b). Interestingly, there was no significant difference in clonality between CD8^+^ PD‐1^HI^ and CD8^+^ PD‐1^LO^ subsets.

### Tumor‐associated antigen‐specific T cells present within the diagnostic tissue can be tracked within the peripheral blood

In viral infections, the clonotype sequences from T cells of known epitope specificity can be used to track T cells over various time points.^17^ In a patient with EBV^+^ DLBCL, we tested whether clonotypes specific to known tumor‐associated antigens can be tracked in tumor, pre‐therapy blood samples and post‐cycle #4 blood samples.

We identified two patients with high *EBER‐1* expression in tumor biopsy. MHC‐I pentamers were used to detect EBV‐specific CD8^+^ T cells in the pre‐therapy blood as previously described.^18^ A sufficient quantity of T cells for downstream TCR sequencing was obtained only from one patient who had type III latency (Supplementary table 2). In that patient, LMP‐2A‐specific CD8^+^ T cells were sorted in the pre‐therapy blood, and the clonotypes were sequenced.

From 15 470 LMP‐2A‐specific CD8^+^ T cells, a total of 1478 productive sequences were obtained, which belonged to 489 unique clonotypes. Of these, 103 clonotypes were present in the diagnostic tumor sample, including 22 clonotypes in the most abundant 100 ITC. These LMP‐2A‐specific CD8^+^ T‐cell clonotypes made up ∼5% of all intratumoral T cells. Of these, six clonotypes were in the most abundant 25 ITC. The number of LMP‐2A‐specific CD8^+^ T‐cell clonotypes detected in the pre‐therapy and post‐cycle #4 peripheral blood repertoires were 284 and 210, respectively (Supplementary figure 1). The LMP‐2A‐specific CD8^+^ T‐cell clonotypes made up 29% of all assayed T cells in the pre‐therapy blood with these clones reducing to 4% of all assayed T cells at the secondary time point.

Next, we analysed the relative abundances of individual LMP‐2A‐specific CD8^+^ T‐cell clonotypes to see whether there were any significant changes during chemoimmunotherapy. To avoid the interferences from extremely low‐frequency clonotypes, this analysis was limited to clonotypes with combined (pre‐therapy + post‐cycle #4) abundances of ≥ 10 productive templates (the threshold for statistical comparison). A difference in clonotype frequencies (*P* < 0.01) was observed in 163 LMP‐2A‐specific CD8^+^ T‐cell clonotypes, of which 162 clonotypes had higher frequency at pre‐therapy time point, one clonotype had higher frequency at the post‐cycle #4 time point, whereas 32 clonotypes did not change during treatment. This indicates that the dynamic changes in tumor antigen‐specific T‐cell clonotypes can be tracked in the peripheral blood, suggesting potential utility as an immune biomarker.

### TCR metrics and intratumoral gene expression

As the majority of abundant intratumoral phenotyped clonotypes were predicted to be CD8^+^ PD‐1^HI^, we tested to see whether there was any correlation between TCR clonotype expansion and expression profile of genes including *CD8*, *PD‐1, LAG3* and *TIM3*. A correlation analysis was performed between TCR clonality, CumFreq‐100, CumFreq‐25 and the normalised gene counts. The correlation coefficient between *CD8* gene count and CumFreq‐25 and Max‐Freq was −0.4 (*P* = 0.04 and 0.03, respectively). The correlation coefficient between *PD‐1* gene count and CumFreq‐100 was −0.3 (*P* = 0.02) and that between *TIM3* gene count and Max‐Freq was −0.4 (*P* = 0.02). Apart from these weak negative correlations, there were no associations between TCR metrics and gene expression profiles, including that of *BCL2, MYC, BCL6* and those associated with COO.

### Intratumoral T‐cell clones are not associated with HLA‐class I genotypes

Given CD8^+^ T cells are likely to contribute to the majority of abundant ITC, we tested for differential expansion of ITC as a function of HLA‐class I genotypes to determine whether there was any relationship between T‐cell clonal expansions, HLA‐class I and iPET result.

There was sufficient DNA for 33 of 35 patients, with 47 class I alleles identified. Homozygosity for HLA‐class I was seen in 10 patients. Thirty‐two alleles were present in more than one patient, and in these patients, the association with TCR metrics and iPET was tested. In multivariate analysis, there was no association between the HLA‐class I genotype and the TCR metrics (productive clonality, CumFreq‐100, CumFreq‐25, Max‐Freq) or with iPET status.

## Discussion

In the current study, samples from a prospective clinical trial of patients with high‐risk DLBCL treated with R‐CHOP had high‐throughput sequencing of the TCRβ‐CDR3 region performed. Our previous work had focused on the TCR repertoire in the tumor tissue, whereas the current study investigated TCR repertoire in tumor and tracked the ITC in sequential blood samples during treatment with R‐CHOP. Analysis of blood samples allowed for a broader depiction of TCR metrics relevant to the whole tumor bulk as opposed to focusing only on the TCR repertoire from a single biopsy site. The primary focus of our study was the TCR repertoire diversity and its relationship with iPET result after four cycles of R‐CHOP which all patients uniformly underwent. As subsequent treatment differed between iPET^+^ and iPET^−^ patients, long‐term clinical outcomes including survival outcomes were not used for analysis. We acknowledge that this is a major limitation of the current study.

We opted to use genomic DNA (gDNA) as it is better validated currently for use with FFPE tissue samples which was the sole tumor tissue source from the study population. Furthermore, gDNA gives better estimate of clonal abundance (number/proportion of T cells with a particular TCR), as there is only one copy of productively rearranged TCR gene per T cell accepting that RNA methods are likely more sensitive.^19^ We employed commercially available ImmunoSEQ (Adaptive Biotech, Seattle, WA, USA) platform, which use multiplex PCR with spike in control for TCR sequencing. The primary metrics we used was productive clonality derived from normalised Shannon's entropy, which has been widely used to report the repertoire diversity.^20^, ^21^ However, like other methods, this method also can be potentially affected by varying sampling depth of real‐life samples. To address this, we have repeated the analysis by downsizing the samples based on the sample with lowest number of TCR read by repeated random sampling as previously described.^22^

Oligoclonal T‐cell expansions within the diagnostic biopsy were predictive of a positive iPET scan. Interrogation of peripheral blood at sequential time points showed that the ITC present within the TME were detectable in the circulation, such that the TCRβ‐CDR3 sequencing was able to track tumor‐associated antigen‐specific T‐cell clones. The most frequent clones found in the tissue biopsy were also frequently found within the peripheral circulation. These ITCs detected in the diagnostic biopsy accounted for approximately 20% of peripheral T cells at diagnosis.

While TCR metrics are widely studied in some solid malignancies where immune‐checkpoint inhibition is frequently the frontline therapy, little is known on the changes that occur in DLBCL patients undergoing chemoimmunotherapy and in particular why at least in the relapse setting, reinvigorating T cells with immune‐checkpoint therapy has been ineffective. The mechanisms underpinning this generally poor response remain unclear.^23^, ^24^ Immune‐checkpoint blockade responsive tumors such as melanoma or mismatch repair‐related colorectal cancers have high baseline T‐cell clonality within their TME.^14^ This is likely because of a higher total mutation burden (TMB), which increases neoantigen production compared to DLBCL.^25^, ^26^ Not only does DLBCL have a lower TMB, but chemoimmunotherapy further reduces the TMB in the vast majority of patients. This may explain the different TCR clonal dynamics observed in DLBCL treated with chemoimmunotherapy compared to solid tumors receiving only immune‐checkpoint blockade. This is consistent with our previous findings that a narrow TCR repertoire is too focused on a select number of tumor‐associated antigens, permitting tumor cells to escape through the gaps in this limited immune ‘net’.^14^ Our findings are further supported by the recent demonstration that a more diverse T‐cell response at baseline as well as increasing diversity of T‐cell clones on therapy was associated with improved outcome in the setting of patients receiving anti‐PD1 therapy for relapsed/refractory Hodgkin lymphoma and in patients with Burkitt lymphoma treated with standard chemotherapy.^27^, ^28^

Within the context of lymphoid malignancy, migration of T cells between the lymphoid and peripheral circulation is of particular interest. However, until now, there had been little data on whether oligoclonal ITC within the TME were present within the blood compartment. Importantly, the demonstration that the oligoclonal ITC were detectable in the blood permitted phenotyping of these shared ITC‐blood clonotypes. The majority (90%) of high‐frequency clones within the TME were reflected in CD8^+^ T cells in the baseline blood, with almost 60% within the CD8^+^ PD‐1^HI^ subsets. Furthermore, overall CD8^+^ T cells in the peripheral blood (including non‐ITC) were significantly more clonal than the circulating CD4^+^ T‐cell pools, which could indicate these CD8^+^ T cells are tumor antigen specific. The negative correlations between *CD8* gene expression and highly clonal T‐cell populations in our study may indicate that these exhausted/dysfunctional clonal T cells may impair the recruitment of further CD8^+^ T cells into the TME. Although we have tracked the ITC in post‐cycle #4 sample, the phenotype especially the PD‐1 status at that time point was not analysed in this study. Further studies are needed to see whether the dynamic changes in the clonotypes abundance and phenotypes at various time points predict treatment response to chemoimmunotherapy or immune‐checkpoint blockades in DLBCL.

In studies on solid organ cancers, clonal tumor‐associated antigen‐specific T cells appear to be enriched within this CD8^+^ PD‐1^HI^ T‐cell compartment.^29^ These CD8^+^ PD‐1^HI^ T cells while considered exhausted still retain the potential for proliferation and cytotoxicity, providing a strong biological rationale for the immune‐checkpoint blockade in malignancy.^30^, ^31^, ^32^, ^33^ Furthermore, there is evidence that peripheral circulating effector T cells, and not those within the TME, mediate the anti‐tumoral effects of the immune‐checkpoint blockade.^34^, ^35^ We have previously shown that fresh CD8^+^ tumor‐infiltrating lymphocytes from DLBCL biopsies demonstrate not only high *PD‐1* expression but also show high co‐expression of other immune checkpoints such as *LAG3* and *TIM3*.^36^ Interestingly, in this study, high *PD‐L1* tumor expression conferred an inferior outcome in those patients, but only in those tumors that also had elevated *LAG3* expression, perhaps suggesting that the exhausted clonal T cells that are expressing multiple immune checkpoints might be inhibiting response to chemoimmunotherapy.^12^

It should be noted that due to the predicted millions of T cells in the circulation of healthy adults, despite the deep sequencing in the peripheral blood that we performed, we cannot exclude that lower frequency but important clones from the tumor may have been missed. In addition, because of the lower numbers of T cells expected to be sequenced from FFPE tissue, clones that might play an important role in anti‐tumor immunity in the periphery could also be missed. Because of the limited availability of paired peripheral blood samples required for extended phenotyping, it was also not possible to comment on the association between T‐cell effector or exhaustion markers in the circulation and PET response.

Individual viral‐specific CD8^+^ T‐cell clones have been previously tracked in the setting of allogeneic stem cell transplantation.^17^ To determine whether high‐throughput sequencing of the TCRβ‐CDR3 region can assist in tracking tumor‐associated antigen‐specific T cells, we interrogated the clonal repertoire of EBV‐specific CD8^+^ T cells from a patient with EBV^+^ DLBCL. We aimed to show that a clone known to have proven anti‐tumor efficacy is present at high levels in the tumor can be found both within the tumor and the periphery and thus may provide insights into our findings in the non‐EBV setting where it is more difficult to prove the specificity of the anti‐tumoral T cells. This patient had a latency III type tumor, which includes expression of LMP‐2A. This analysis showed that the LMP‐2A‐specific CD8^+^ T cells had undergone large clonal expansions that were shared between the tumor tissue and blood and that these could be tracked during therapy. LMP‐2A‐specific CD8^+^ T‐cell clones accounted for almost 25% of the total circulating T cells at diagnosis. Interestingly, this level of EBV‐specific T‐cell clonal immunity is similar to that described in the setting of infectious mononucleosis, and it is known that *PD‐1* is highly expressed on LMP‐2A‐specific CD8^+^ T cells in acute infection.^37^ This may support our assumption that ITCs found in the circulation could well be tumor‐specific, but further studies using other tumor antigens will be required to confirm this. Furthermore, the dynamic changes in clonotypes between time points with many clones disappearing at the secondary time point were broadly consistent with a convalescent viral state.^38^, ^39^, ^40^ Similarly, ∼20% of the deeply sequenced peripheral blood TCRs in other patients were clones found in the tumor. Further study will be required to understand whether these clones are directed against lymphoma or even clones directed against other common antigens present at high levels throughout the body. Our results must not be over‐interpreted given the sample size and issues related to PET scan interpretation and outcome, and further tracking studies are required to assess the importance of dynamic shifts in T‐cell clones on tumor control. Clonality in the peripheral blood at diagnosis or post‐4 cycles R‐CHOP did not correlate with PET scan results.

CD8^+^ T cells recognise immunogenic peptides in the context of HLA class I. It has previously been demonstrated that the patients with specific HLA class I genotypes are less susceptible to the development of EBV^+^ Hodgkin lymphoma.^41^ We have previously shown that this is likely explained by the increased frequency of EBV‐specific CD8^+^ T‐cell clones observed in patients with these HLA alleles.^42^ However, the relationship between T‐cell clonality and HLA‐class I remains untested in other lymphomas. In this cohort of high‐risk DLBCL, HLA‐class I genotype was not found to be associated with productive clonality, CumFreq‐100, CumFreq‐25 or iPET status.

The importance of TCR clonal repertoire in predicting PET response is likely to have significance more as a biomarker of specific T‐cell responses rather than as a stand‐alone biomarker of response. It is unlikely to outperform new technologies such as circulating tumor DNA as a disease response biomarker; however, it is vital to understand why a clonal T‐cell population is predicting for poor outcome. A better understanding of this phenomenon will be important in improving outcomes with therapies such as CAR‐T and other immune‐based therapies that depend on T‐cell responses and/or activation. Future studies should also address TCR repertoire changes based on mutational classification of the DLBCL tumor and double‐hit status. In addition, information on TCR clonality from different tumor sites in the same patient (and potentially changes in an on‐treatment biopsy and/or relapse) and how this influences TCR metrics in the peripheral blood and response is not yet determined but will likely influence the future of T‐cell‐derived therapies.

In conclusion, restriction of the TCR repertoire to a small number of highly expanded T‐cell clones within the TME of high‐risk DLBCL appears to predict an inferior outcome with conventional frontline chemoimmunotherapy. Oligoclonal ITC were present in the peripheral circulation and were predominantly CD8^+^ PD‐1^HI^. Tumor‐associated antigen‐specific T cells present within the diagnostic tissue (in an EBV‐associated case) could be tracked within the peripheral blood, indicating their potential utility as biomarkers of response to novel immunotherapies. Further studies are required to see whether these dominant exhausted T‐cell populations can be manipulated and or replaced by more active T cells to enhance responses in DLBCL.

## Methods

### ALLGNHL21 trial design and patient selection for the current study

The ALLGNHL21 trial (ACTRN 12609001077257) was a multicentre phase II trial, exploring the role of iPET guided treatment intensification in high‐risk DLBCL treated with R‐CHOP. Patients aged 18–70 years with high‐risk DLBCL (either IPI 2–5 or 0–1 with most > 7.5 cm), who were previously untreated, were considered fit for autologous stem cell transplantation, and who had a positive baseline PET scan with more than one FDG‐avid lesion were enrolled in the study.^43^ The iPET was performed after four cycles of R‐CHOP and was read centrally. The iPET^+^ patients underwent significant treatment intensification with chemoimmunotherapy followed by autologous stem cell transplantation, whereas the iPET^−^ patients completed six cycles of R‐CHOP. The participants had provided written informed consent for biobanking of formalin‐fixed paraffin‐embedded (FFPE) tumor biopsies and PBMCs collected at pre‐therapy and post‐cycle #4. Based on the availability of these tissues, 35 patients were selected for further analysis. All 35 patients had tumor biopsy samples available, and among these, 24 patients had paired pre‐therapy and post‐cycle #4 PBMC samples available. Among the remaining patients, six patients had only pre‐therapy PBMC samples, three patients had only post‐cycle #4 samples, and two patients had no PBMC samples available. This study was approved by the responsible human research ethics committees.

### TCR sequencing of tumor and peripheral blood

Genomic DNA (gDNA) was extracted from FFPE tumor biopsies and cryopreserved PBMC. The TCRβ‐CDR3 was amplified using the *ImmunoSEQ hsTCRB* kit (Adaptive Biotechnologies). When available, at least 1000 ng of gDNA was used for tumor samples and 2500 ng for PBMC samples (aiming for 500 ng per PCR). Based on the expected T‐cell content, the tumor samples were analysed in replicates of two (survey level sequencing) and PBMC in replicates of five (deep level sequencing). T‐cell sequencing yield from FFPE biopsies were substantially lower than that from PBMC which is reflected in the difference in the sequencing depth between PBMC and tumor tissue. The pooled libraries were sequenced on Illumina Inc. San Diego, CA, US NextSEQ‐500 analyser. The raw sequencing data were uploaded to the *ImmunoSEQ* pipeline for processing.

### Analysis of TCR data

TCR data were analysed as previously outlined.^14^ Briefly, the assay employed spike‐in controls to detect primer bias and amplification of individual samples, from which the starting number of CDR3 sequences (templates) present in each sample was calculated. Each unique CDR3 nucleotide sequence was termed a clonotype. From the total number of templates and unique clonotypes, the productive clonality (a measure of lack of diversity) of the samples was calculated utilising Shannon's entropy as previously described. Additionally, the productive clonality was repeatedly calculated after normalising the samples by repeated (*n* = 5) random drawing of repertoire based on the lowest number of productive templates in the analysis (*n* = 603). On a scale of 0 to 1, a value close to 0 was indicative of a highly diverse TCR repertoire, whereas a value close to 1 was indicative of extreme loss of diversity because of oligoclonal expansion. Additional measures of clonality (that specifically measure oligoclonal expansions) tested were the cumulative frequencies of the most abundant 100 clonotypes (CumFreq‐100) and the cumulative frequencies of the most abundant 25 clonotypes (CumFreq‐25). For downstream analysis involving tracking of clonotypes within individual patients, CDR3 nucleotide sequences were used for more precise tracking.

### FACS sorting of peripheral blood mononuclear cells

To establish the phenotype of shared ITC in peripheral blood, pre‐therapy PBMC from six patients were sorted into seven populations and TCR sequencing performed as follows. These patients were selected based on iPET results (three iPET^+^; three iPET^−^), their high level of CDR3 sequence sharing between tumor and peripheral blood, and the availability of sufficient samples for further analysis.

Cryopreserved PBMC were thawed in a 37°C water bath until a small clump of ice left. The cells were then transferred to pre‐warmed GIBCO^TM^RPMI1640 (Thermo Fisher Scientific, Waltham, MA, US) containing 1% penicillin, streptomycin and glutamate (PSG) and 1% sodium pyruvate (hereafter referred to as complete RPMI). Cells were centrifuged down and resuspended with 10 mL of 10% heat‐inactivated foetal bovine serum (FBS)/complete RPMI. Cells were rested in 37°C incubator for 30 min. Live cells were determined by trypan blue exclusion. Cells were centrifuged down, resuspended with ice‐cold 1× FACS wash (1× PBS/ 0.1% BSA/2mM EDTA) and stained with CCR7 at room temperature for 15 min to get a better separation. The remaining surface markers were added to the cells and incubated on ice for another 20 min. Cells were then washed with 1× FACS wash once and 1× PBS once. Cells were resuspended with 1× PBS and stained with Live/Dead Fixable Near‐IR Dead Cell Stain (Invitrogen) for 10 min on ice. Cells were then washed twice with 1× FACS wash to stop the staining. Cells were resuspended with 1× FACS wash at 10 million cells mL^−1^, filtered through a 70‐µm filter (BD Biosciences, San Jose, CA, US) and sorted on the BD FACS Aria Fusion sorted (BD Biosciences) into seven populations: (1) CD8^+^ PD‐1^HI^, (2) CD8^+^ PD‐1^LO^, (3) CD4^+^ PD‐1^HI^, (4) CD4^+^ PD‐1^LO^, (5) CD4^+^ T effector memory cells, (6) CD4^+^ T‐regulatory cells and (7) the remaining CD4^+^ cells (comprising CD4^+^ T central memory cells, Temra and naïve cells). Please see the Supplementary data for the antibodies used (Supplementary table 3) and the gating strategy (Supplementary figure 2). Sorted cells were collected in LoBind DNA tubes (Eppendorf), cells were washed with 1x PBS, and the cell pellets were stored at −80°C until DNA extraction. The gDNA from sorted populations was subjected to TCR sequencing in replicates of two, and the clonotypes were matched with clonotypes from (unsorted) DLBCL diagnostic biopsies. The phenotypes of individual ITC were then inferred from the phenotypes of the shared clonotypes in the peripheral blood.

### HLA‐class I genotyping and analysis

The genotypes of chromosome 6 were extracted and then imputed against *1000G Phase 3 v5* reference by the Michigan Imputation Server, to obtain input data for submission to the HLA*IMP:03 web server. The final HLA‐class I imputation was completed by HLA*IMP:03 web server.

### Gene expression profiling

RNA from FFPE samples was extracted using Ambion^TM^ *RecoverAll* ^TM^ total nucleic acid extraction kit for FFPE (Thermo Fisher Scientific, Waltham, MA, US) as per the manufacturer's instructions. As previously published, a custom panel of immune genes was quantified using the *nCounter* platform (nanoString Technologies), and the cell of origin (COO) was calculated by *Lymph2Cx*.^11^, ^44^

### Pentamer sorting of EBV epitope‐specific T cells

To test whether known tumor‐associated antigen‐specific T cells could be tracked in peripheral blood, we employed EBV‐specific CD8^+^ T cells from patients with EBV^+^ DLBCL. The EBV‐latency profiles of patients were established as described before.^45^ Based on their EBV‐latency profile and HLA‐I types, MHC pentamers were chosen (ProImmune Ltd, Oxford, UK) and sorting was performed on pre‐therapy blood. TCR sequencing was performed on these cells as mentioned above.

### Statistical methods

Unless indicated otherwise, the continuous variables were described as median (range) and groups were compared with the Mann–Whitney *U‐*test, Kruskal–Wallis test or Wilcoxon ranked sum test as appropriate. Regression analyses were performed on R Statistical Computing environment (R Foundation for Statistical Computing, Vienna, Austria. URL http://www.R‐project.org) using EZR on R commander.^46^ All other statistical tests were performed on GraphPad Prism for macOS version 8.4.3.

## Conflict of Interest

CK has received consultancy fees, honoraria and or research funding from Bristol‐Myers Squibb, Celgene, Gilead Sciences, Janssen‐Cilag, MSD Oncology and Roche. MKG has received consultancy fees, honoraria and or research funding from Amgen, Bristol‐Myers Squibb, Celgene, Genentech, Gilead Sciences, Janssen‐Cilag, Merck Sharp & Dohme and Roche. JFS has received consultancy fees, honoraria and or research funding from AbbVie, Astra Zeneca, Celgene, Genentech, Gilead Sciences, Janssen‐Cilag, Mei Pharma, Morphosys, Roche, Sunesis and Takeda. The remaining authors declare no competing financial interests.

## Author contributions

MS, MKG and CK designed and performed the research, interpreted the results and wrote the manuscript; MS, S‐CL, MBS, LML, KN and JG performed the experiments; ZL performed the HLA imputation; RJH interpreted the iPET scans; MS analysed the data; MH, RJH, JFS, MKG designed and conducted the ALLGNHL21 trial; and all authors were involved in the development and final approval of the submitted manuscript.

## Supporting information

Supplementary Material

## Data Availability

The TCR sequencing data included in this report are deposited in the immuneACCESS repository, and this can be accessed at *immunoSEQ*.
