# Supplementary material for: Intratumoral T‐cell receptor repertoire is predictive of interim PET scan results in patients with diffuse large B‐cell lymphoma treated with rituximab/cyclophosphamide/doxorubicin/prednisolone/vincristine (R‐CHOP) chemoimmunotherapy
Source: Clin Transl Immunology. 2021 Oct 27;10(11):e1351. doi: 10.1002/cti2.1351 (PMC8548874; doi:10.1002/cti2.1351)
Supplement: Supplementary file 1 — Supplementary Material [file CTI2-10-e1351-s001.pdf]

Supplementary table 1

TCR sequencing results of pre-therapy peripheral blood T-cell populations sorted by FACS in selected patients (n=6).

T-cell populations	Yield (number of cells)	TCR sequencing results				
		DNA input ng	Total productive sequences	Unique productive sequences	Productive clonality	MaxFreq
$CD4^+$ PD-1^{HI}	14,525 (4,740-33,060)	64 (34-220)	3,108 (669-6,908)	2,604 (607-5,832)	0.01 (0.005-0.014)	0.005 (0.002-0.02)
$CD4^+$ PD-1^{LO}	10,545 (3,860-37,150)	52 (6-288)	527 (121-10,925)	468 (103-8,342)	0.008 (0.007-0.14)	0.01 (0.001-0.033)
Tregs	28,465 (12,030-100,000)	151 (90-216)	7,499 (1,116-18,727)	5,353 (967-13,056)	0.022 (0.007-0.045)	0.005 (0.003-0.015)
CD4-Tem	27,485 (5,130-58,330)	80 (9-964)	2,044 (42-15,607)	935 (37-7,989)	0.055 (0.011-0.236)	0.051 (0.005-0.219)
Other $CD4^+$T cells	145,500 (48,040-290,000)	292 (122-426)	19,551 (8,298-45,252)	17,369 (7,208-38,270)	0.005 (0.003-0.008)	0.001 (0.0-0.001)
$CD8^+$ PD-1^{HI}	89,545 (29,220-153,000)	316 (129-595)	20,659 (1,926-37,129)	2,630 (743-6,009)	0.292 (0.202-0.604)	0.134 (0.063-0.534)
$CD8^+$ PD-1^{LO}	56,945 (12,480-223,000)	100 (69-346)	11,085 (290-24,619)	1,564 (228-4,524)	0.340 (0.032-0.704)	0.116 (0.033-0.711)

MaxFreq, frequency of most abundant clonotype in the sample; Tregs, $CD4^+$ regulatory T cells ($CD4^+CD25^{HI}CD127^{Lo}$); CD4-Tem, CD4-effector memory cells ($CD4^+CCR7^{-ve}CD45RA^{-ve}$).

All values are median (range).

Supplementary table 2

Yield of EBV-specific T cells by pentamer sorting in 2 patients with high EBER-1 expression (EBER-1 gene count >1000) in the diagnostic biopsy.

Patient	Age, Sex	EBV-latency profile	MHC-I pentamer	Epitope Target	T cell yield (n)
01	53, F	Latency III	A*24:02-TYG	LMP-2	15,470
			A*02:01-FLY	LMP-2	19
02	57, F	Latency I	B*35:01-HPV	EBNA-1	260

EBER, EBV-encoded RNA; LMP, EBV-latent membrane protein; EBNA, EBV-nuclear antigen.

Supplementary table 3

Antibodies used for FACS

Antibody	Clone	Fluorochrome	Manufacturer	Isotype control
CD14	M5E2	FITC	BD Biosciences	Mouse IgG2a, k
CD16	3G8	FITC	Biolegend	Mouse IgG1, k
CD19	HIB19	FITC	BD Biosciences	Mouse IgG1, k
CD56	AF12-7H3	Vio-Bright FITC	Miltenyi Biotec	Mouse IgG1, k
PD-1	PD1.3.1.3	PE	Miltenyi Biotec	Mouse IgG2b, k
CD127	hIL-7R-M21	PerCP/Cy5.5	BD Biosciences	Mouse IgG1, k
CD25	BC96	APC	Biolegend	Mouse IgG1, k
CD45RA	HIB100	Alexa Fluor700	BD Biosciences	Mouse IgG2b, k
CCR7	150503	BV421	BD Biosciences	Mouse IgG2a, k
CD8	SK1	BV785	Biolegend	Mouse IgG1, k
CD3	SK7	BUV395	BD Biosciences	Mouse IgG1, k
CD4	SK3	BUV737	BD Biosciences	Mouse IgG1, k
Live/Dead Fixable Near-IR Dead Cell Stain	-	-	Invitrogen	-

Supplementary figure 1

Tracking the EBV-specific CD8⁺ T-cell clonotypes in diagnostic biopsy, pre-therapy blood and post cycle #4 blood samples.

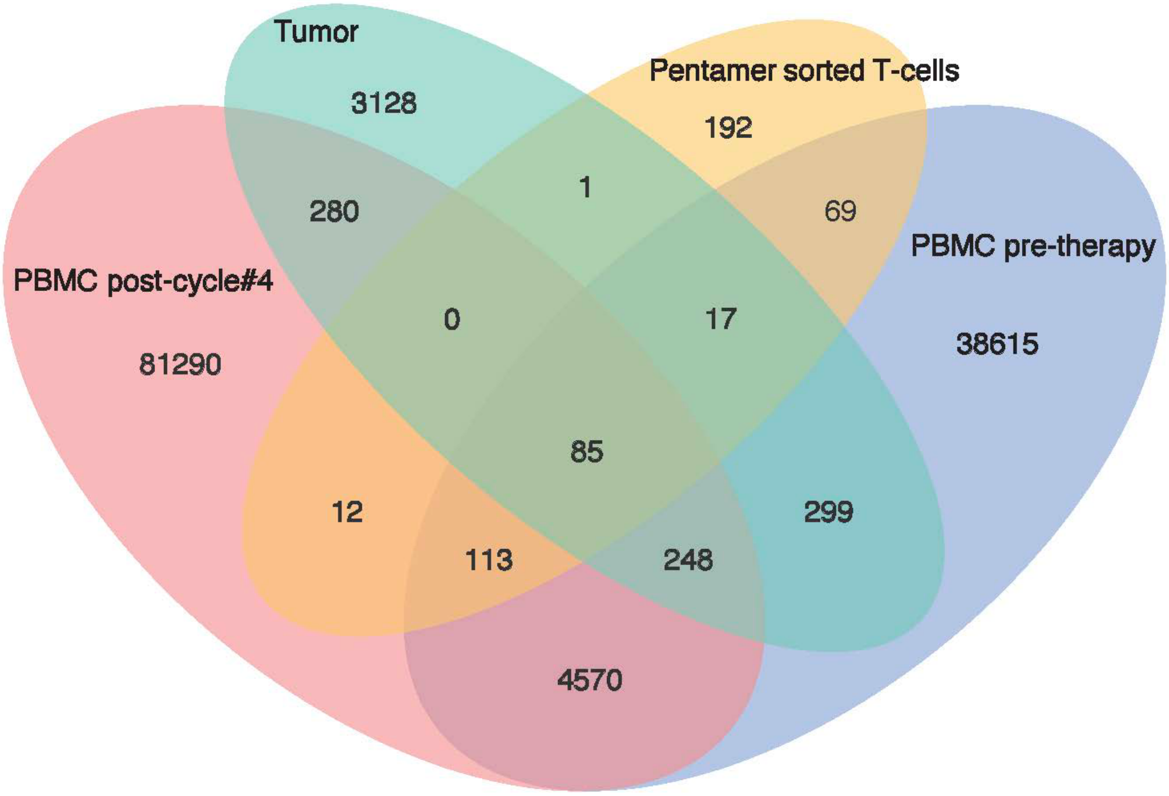

EBV-specific CD8$^+$ T-cell clonotypes (targeting LMP-2) were sorted by HLA- A*24:02-TYG pentamers in a patient with EBV$^+$ DLBCL. These clonotypes were tracked in diagnostic biopsy and pre-therapy and post-cycle #4 blood samples by CDR3 nucleotide matching. The numbers shown are those of unique clonotypes.

Supplementary figure 2

FACS Gating strategy

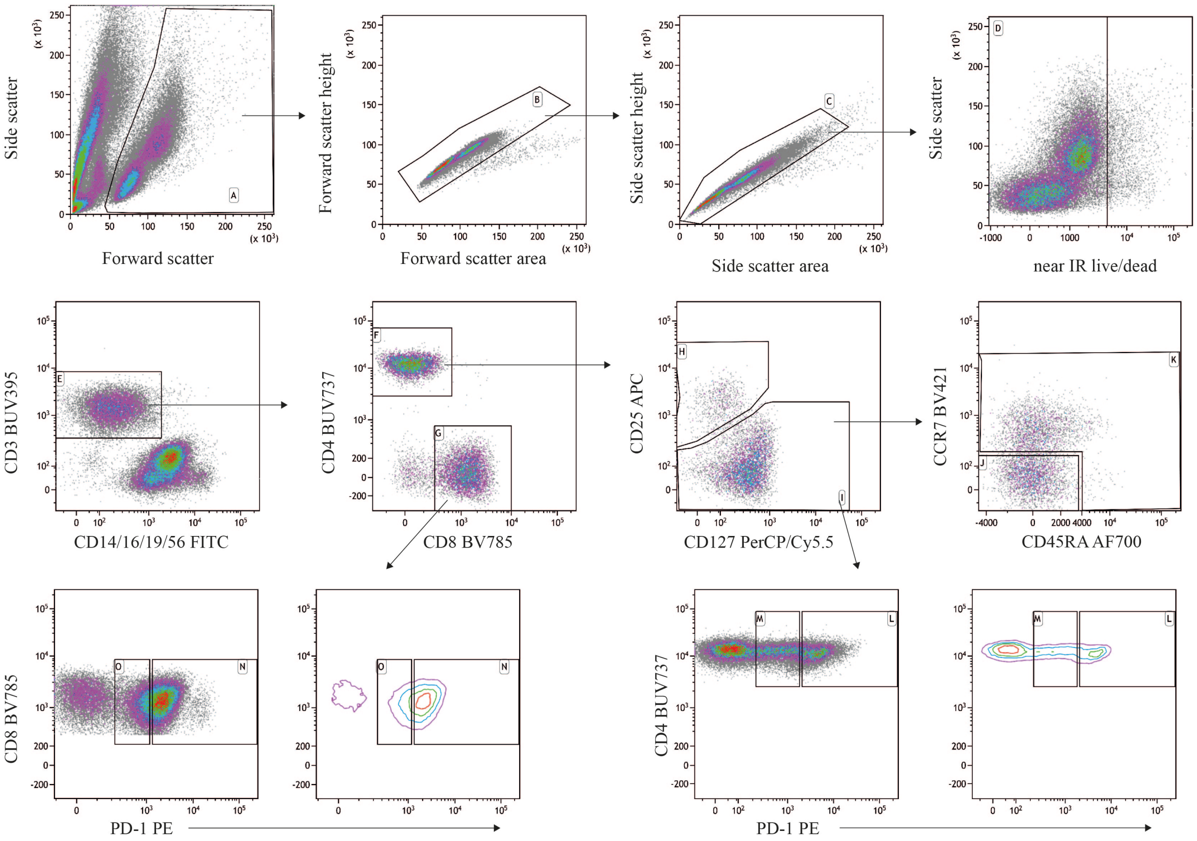

Flow cytometry gating strategy for T-cell subpopulations: Live singlet lymphocytes were gated. Viable cells were selected by LIVE/DEAD™ Fixable Near-IR Dead Cell Stain. T cells were identified by CD3+ CD14- CD16- CD19- CD56-. $CD3^+$ T cells were then separated in to CD4 and CD8. $CD8^+$ T cells were sorted into 2 populations based on their PD-1 expression: $CD8^+$ PD-1^{HI}, and $CD8^+$ PD-1^{LO}. $CD4^+$ T cells were first sorted into $CD25^{HI}$ $CD127^{LO}$ Treg and $CD25^{LO}$ $CD127^{HI}$ T cells. The $CD25^{LO}$ $CD127^{HI}$ subset was further sorted into $CD4^+$ effector memory T cells ($CCR7^{-ve}$ $CD45RA^{-ve}$), combined $CD4^+$ central memory, naive and CD45RA expressing effector memory T cells, $CD4^+$ PD-1^{HI} and $CD4^+$ PD-1^{LO} T cells.
